# Trends and Inequities in Use of Maternal Health Care Services in Bangladesh, 1991-2011

**DOI:** 10.1371/journal.pone.0120309

**Published:** 2015-03-23

**Authors:** Iqbal Anwar, Herfina Y. Nababan, Shabnam Mostari, Aminur Rahman, Jahangir A. M. Khan

**Affiliations:** 1 International Centre for Diarrheal Disease Research (icddr,b), Dhaka, Bangladesh; 2 James P. Grant School of Public Health, BRAC University, Dhaka, Bangladesh; University of South Carolina, UNITED STATES

## Abstract

**Background and Methods:**

Monitoring use-inequity is important to measure progress in efforts to address health-inequities. Using data from six Bangladesh Demographic and Health Surveys (BDHS), we examine trends, inequities and socio-demographic determinants of use of maternal health care services in Bangladesh between 1991 and 2011.

**Findings:**

Access to maternal health care services has improved in the last two decades. The adjusted yearly trend was 9.0% (8.6%-9.5%) for any antenatal care (ANC), 11.9% (11.1%-12.7%) for institutional delivery, and 18.9% (17.3%-20.5%) for C-section delivery which is above the WHO recommended rate of 5-15%. Use-inequity was significant for all three indicators but is reducing over time. Between 1991-1994 and 2007-2011 the rich:poor ratio reduced from 3.65 to 1.65 for ANC and from 15.80 to 6.77 for institutional delivery. Between 1995-1998 and 2007-2011, the concentration index reduced from 0.27 (0.25-0.29) to 0.15 (0.14-0.16) for ANC, and from 0.65 (0.60-0.71) to 0.39 (0.37-0.41) for institutional delivery during that period. For use of c-section, the rich:poor ratio reduced from 18.17 to 13.39 and the concentration index from 0.66 (0.57-0.75) to 0.47 (0.45-0.49). In terms of rich:poor differences, there was equity-gain for ANC but not for facility delivery or C-section delivery. All socio-demographic variables were significant predictors of use; of them, maternal education was the most powerful. In addition, the contribution of for-profit private sector is increasingly growing in maternal health.

**Conclusion:**

Both access and equity are improving in maternal health. We recommend strengthening ongoing health and non-health interventions for the poor. Use-inequity should be monitored using multiple indicators which are incorporated into routine health information systems. Rising C-section rate is alarming and indication of C-sections should be monitored both in private and public sector facilities.

## Introduction

The continuing use of maternal mortality ratio (MMR) as an outcome indicator for tracking the progress of Safe Motherhood faces many challenges [1]. Process indicators, however, provide more information for day-to-day monitoring and measure the use of maternal health care services most likely to impact MMR [2]. In addition, inequity has been identified as a key constraint in maternal health [3, 4], and existing data confirms significant inequities between and within countries with regards to both process and outcome indicators [3, 5]. A rapid increase in interest at the policy level has resulted in many innovative demand-side health financing strategies being implemented in different contexts [6]. The routine monitoring of use-inequity is therefore important in monitoring the progress of these pro-poor health financing strategies.

For the past two decades, Bangladesh has been implementing evolving maternal health strategies to address its high maternal mortality [7–9]. In 1994, the Ministry of Health and Family Welfare (MOHFW) initiated the upgrade of peripheral level public health facilities in providing basic and comprehensive emergency obstetric care (EmOC) services in a phased manner [10]. A home-based skilled birth attendants training program was initiated in 2003 as well as a demand side financing (DSF) maternal health voucher scheme in 2007 to increase the utilization of maternal health care services by the poor [11]. Despite these efforts, the progress was slow in improving access to care by all segments of the community [10]. Care-seeking is a complex behavioral phenomenon shaped by both supply and demand side factors. Improving supply is important, but it is not always sufficient to improve access [8, 12]. Hence, there is a need to understand the role of socio-demographic factors in determining the use of maternal health care services.

In Bangladesh, a number of studies have explored high levels of use-inequity in maternal health by wealth quintile, maternal education and urban versus rural locations [7,10, 13–16]. However, these studies were largely small scale, conducted in single communities, using single year data, inhibiting consideration of trends in inequities in use of maternal health care services. More importantly, these studies used indicators such as equity gaps, equity ratios and odds ratios of use rather than state-of-the-art methods such as concentration index for measuring use-inequities. Demographic and Health Surveys (DHSs) serves as one of the main data sources for monitoring trends and inequities in the use of maternal health care services due to the quality of the data and comparability over time and between countries [1]. In this study, we analyzed data from six consecutive Bangladesh Demographic and Health Surveys (BDHSs) to explore trends and inequities in antenatal care (ANC) uptake, facility delivery and Caesarean section (C-section) delivery in an effort to inform post-MDG strategies and policies for ending preventable maternal mortality.

## Methods and Materials

With the permission of the MEASURE DHS [Monitoring and Evaluation to Assess and Use Results, Demographic and Health Surveys] (www.measuredhs.com), the 1993–1994, 1996–1997, 1999–2000, 2004, 2007 and 2011 BDHS data-sets were downloaded, merged and analyzed to look into trends, inequities and other socio-demographic determinants of use of maternal health care services. This study was exempt from review by the Human Investigation Committee (HIC) as publicly available BDHS data was used and no identifying participant information was obtained. All data was reported in aggregate and no attempts were made to identify individual study participants.

The outcome measure focused on whether women received ANC, whether they had institutional deliveries and whether they delivered by caesarean-section (C-section); while the exposure (predictor) variables used in the analysis included asset quintile, mother’s and father’s highest level of education (none, primary, secondary, higher), area of residence (urban/rural), religion (Islam/Hindu and others), maternal age (low-19, 20–29, 30–39 and, 40 and above), birth order (1, 2, 3, 4, 5, 6+), year of birth (in four year groups), and the administrative division (Barisal, Chittagong, Dhaka, Rajshahi, Sylhet, Khulna and Rangpur). Asset quintiles were computed from the asset variables present in each survey using the statistical methods of Principal Components and Factor Analysis [17, 18]. Asset quintiles were generated separately for each survey and the asset variables included: source of drinking water, type of latrine, principal construction material of floor, wall, and roof, electricity supply, and ownership of radio, television and bicycle. Asset quintiles were derived from the first principal component. Trends in socio-demographic variables were examined by analyzing their changes over 4-year time periods except for 2007–2011 which covered a period of 5 years.

Inequities were measured in terms of asset quintile, area of residence and parental education. To look into trends in use-inequities, we used rich:poor ratio, rich:poor difference and the concentration index (with 95% confidence intervals) based on asset quintiles [19, 20]. We estimated the concentration index for all three outcome variables with their 95% confidence intervals. This concentration index quantifies how specific goods and services are distributed across socioeconomic groups. To calculate this index, a concentration curve was constructed where the cumulative proportion of population (for instance, target population who are in need of institutional delivery) is plotted against the cumulative proportion of services (institutional delivery) after ranking the population from the lowest to the highest socioeconomic status considering asset index. The concentration index is defined as twice the area between the concentration curve and the line of equality. The index varies between +1 and -1. Positive values indicate a higher concentration of services in higher socioeconomic groups and vice-versa. If there is no socioeconomic-related inequality, the concentration index is zero. While regression analysis shows how one variable (e.g. socioeconomic status) influences the dependent variable (e.g. received institutional delivery), the concentration index provides the relative measurement of the distribution. Concentration indices, rich:poor ratios and rich:poor differences for ANC, institutional delivery and C-section use were compared over time periods to comment on trends in inequities in the use of maternity care services.

Binary logistic regression models were developed to obtain crude and adjusted odds ratios for each outcome variable using Wald tests to assess statistical significance, taking into account survey design (sampling weights and strata) and clustering (to account for women who contributed more than one live-birth in one in the preceding 3–5 years). Forward stepwise method in binary logistic regression models in SPSS 20 (IBM Corporation, Chicago, IL) was used to look for the socio-demographic predictors by order of their strength of association with the outcome variables. Time trends were examined by asset quintile and area of residence (urban versus rural) to determine whether the odds of use (ANC, facility delivery and delivery by C-section) were changing at the same rate in all socio-economic groups and in urban and rural areas.

## Results

### Trends in socio-demographic characteristics of the population

Our initial analysis included 38,706 women who had given birth between 1991 and 2011. However, information on C-sections was absent in the first two surveys (BDHS 1993–1994 and BDHS 1996–1997), and there were missing values for some of the exposure and outcome variables. As a result, the number (N) varied throughout the analysis. Trend data clearly showed that there have been considerable socio-demographic changes in Bangladesh over the last two decades. The percentage of mothers with no education decreased from 58% in 1991–1994 to 20.1% in 2007–2011 and from 48.2% to 29.6% for their husbands (Table 1). There was little change in the age distribution of mothers. However, the rates of teenage pregnancy remained high throughout the reporting period. There was a major shift in the distribution of birth order of mothers; between 1991–1994 and 2007–2011 the proportion of women with first birth order increased from 18.2% to 29.0% while women with birth-order six and above reduced from 10.9% to 2.6% (Table 1).

**Table 1:** Trends in socio-demographic characteristics in Bangladesh (1991–2011).

Socio-demographic	1991–1994	1995–1998	1999–2002	2003–2006	2007–2011	P value
variables	(N-7608)	(N-7589)	(N = 7407)	(N = 7073)	(N = 8759)	
**Maternal education**						
No education	57.9	50.9	40.3	28.2	20.1	p<0.001
Primary	27	28.3	30.7	31.4	30.7	
Secondary	13.1	17.6	24.7	34.6	42	
Higher	1.9	3.2	4.3	5.8	7.2	
**Paternal education**						
No education	48.6	45.9	40.9	36.5	29.6	p<0.001
Primary	24.5	25	27.3	28	29	
Secondary	19.7	20.4	23.2	24.9	28.9	
Higher	7.2	8.7	8.5	10.6	12.4	
**Area of residence**						
Rural	90.5	85.7	80.9	80	77.3	p<0.001
Urban	9.5	14.3	19.1	20	22.7	
**Age group of the mother**						
10–19	14	17.3	14.7	17.8	15	p<0.001
20–29	61	57.9	59	60.2	63.5	
30–39	21.6	21.6	23	19.6	19	
≥40	3.4	3.2	3.2	2.5	2.5	
**Religion**						
Islam	90.1	90	91.7	92.3	91.4	p<0.001
Hindu and others	9.9	10	8.3	7.7	8.6	
**Birth order**						
1	18.2	21.4	20.2	26.8	29	p<0.001
2	23.9	26.4	27.4	28.7	31.7	
3	18.6	18.7	19.9	18.3	19	
4	13.2	12	12.9	11.5	10	
5	8.6	7.6	7.7	6.4	4.9	
6 and above	17.5	13.9	11.8	8.3	5.3	

### Trends in use of maternal health care services

**Fig 1:**
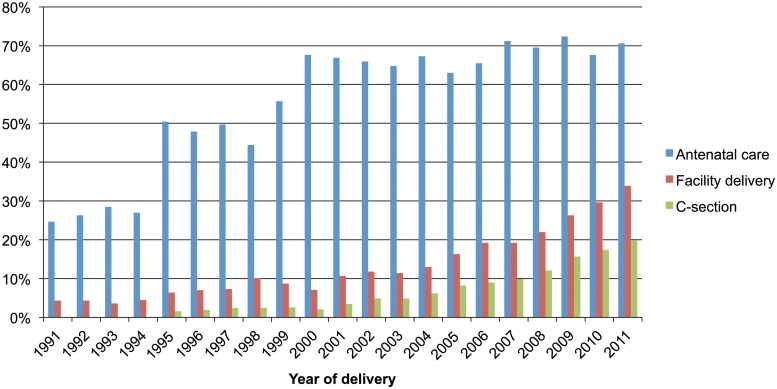
Trend in use of ANC, facility delivery and C-section1991–2011.

Fig. 1 illustrates that between 1991 and 2011, the percentage of mothers with at least one ANC consultation increased from 24.7% to 70.6% and the institutional delivery rate increased from 4.3% to 33.9%. Meanwhile, the population based C-section rate increased from 1.6% in 1995 to 19.8% in 2011. The rates of annual increase were statistically significant for all three use variables (Table 2). However, the rate of annual increase was more for C-section delivery than for institutional delivery or use of any ANC services. Adjusted yearly trend was 9.0% (OR 1.090; 95% CI of OR 1.086–1.095) for use of any ANC; 11.9% (OR 1.119; 95% CI of OR 1.111–1.127) for institutional delivery; and 18.9% (OR 1.189; 95% CI of OR 1.173–1.205) for C-section delivery. A women who delivered during 2007–2011 were 5.16 (95% CI 4.58–5.80) times more likely to receive ANC services and 6.11 (95% CI 5.10–7.32) times more likely to deliver in health facilities than women who delivered during 1991–1994 after adjusting for all socio-demographic covariates in the model. Similarly, women who delivered during 2007–2011 were about 6.37 (95% CI 5.01–8.08) times more likely to deliver by C-section than women who delivered during 1995–1998 (Table 3).

**Table 2:** Annual trends in maternity care indicators in Bangladesh (1991–2011) stratified by asset quintile and area of residence.

	Annual Trends					
	Any ANC		Institutional delivery		C-section	
	Univariate	Multivariate	Univariate	Multivariate	Univariate	Multivariate
	OR	OR	OR	OR	OR	OR
	(95% CI)	(95% CI)	(95% CI)	(95% CI)	(95% CI)	(95% CI)
**Overall Yearly Trend**	1.109	1.09	1.134	1.119	1.201	1.189
	(1.105–1.113)	(1.086–1.095)	(1.128–1.141)	(1.111–1.127)	(1.187–1.215)	(1.173–1.205)
**Asset quintile**						
Poorest	1.096	1.087	1.141	1.12	1.218	1.196
	(1.088–1.105)	(1.078–1.096)	(1.117–1.166)	(1.094–1.147)	(1.148–1.293)	(1.122–1.274)
Poor	1.085	1.066	1.16	1.132	1.313	1.282
	(1.077–1.094)	(1.057–1.076)	(1.138–1.182)	(1.108–1.156)	(1.255–1.373)	(1.221–1.346)
Middle	1.129	1.095	1.187	1.138	1.3	1.266
	(1.120–1.138)	(1.085–1.106)	(1.167–1.207)	(1.116–1.160)	(1.253–1.350)	(1.215–1.318)
Richer	1.15	1.11	1.192	1.156	1.258	1.231
	(1.139–1.160)	(1.098–1.121)	(1.176–1.209)	(1.139–1.174)	(1.224–1.293)	(1.195–1.268)
Richest	1.153	1.104	1.19	1.093	1.119	1.143
	(1.139–1.166)	(1.089–1.119)	(1.109–1.130)	(1.081–1.105)	(1.109–1.130)	(1.122–1.164)
**Area of residence**						
Rural	1.108	1.094	1.156	1.141	1.243	1.232
	(1.104–1.112)	(1.089–1.099)	(1.147–1.165)	(1.131–1.151)	(1.224–1.264)	(1.210–1.255)
Urban	1.072	1.063	1.077	1.083	1.44	1.139
	(1.061–1.083)	(1.050–1.076)	(1.067–1.088)	(1.070–1.097)	(1.124–1.164)	(1.117–1.163)

**Table 3:** Determinants of antenatal care, institutional delivery and c-section in Bangladesh (1991–2011).

	Antenatal care			Institutional delivery			Cesarean section		
	(N = 32,619)			(N = 38,438)			(N = 28,468)		
	% use	Univariate	Multivariate	% use	Univariate	Multivariate	% use	Univariate	Multivariate
		OR (95% CI)	OR (95% CI)		OR (95% CI)	OR (95% CI)		OR (95% CI)	OR (95% CI)
**Year groups**									
1991–1994	27.2%	1	1	4.1%	1	1.00			
1995–1998	34.9%	2.55 (2.38–2.72)	2.67 (2.41–2.96)	8.1%	1.87 (1.62–2.15)	1.76 (1.47–2.11)	2.6%	1	1
1999–2002	52.8%	4.66 (4.35–4.99)	4.70 (4.18–5.28)	11.3%	2.47 (2.15–2.83)	2.13 (1.77–2.56)	4.1%	1.55 (1.24–1.94)	1.52 (1.17–1.96)
2003–2006	61.1%	5.09 (4.75–5.47)	4.44 (3.93–5.01)	16.7%	3.93 (3.45–4.48)	3.04 (2.53–3.65)	7.8%	3.30 (2.69–4.05)	2.83 (2.22–3.61)
2007–2011	67.4%	6.43 (6.01–6.89)	5.16 (4.58–5.80)	27.7%	7.86 (6.95–8.88)	6.11 (5.10–7.32)	15.2%	7.64 (6.3–9.27)	6.37 (5.01–8.08)
**Asset quintile**									
Poorest	31.4%	1	1	4.0%	1	1.00	1.2%	1	1
Poor	34.6%	1.21 (1.14–1.29)	1.05 (.99–1.26)	4.1%	1.45 (1.26–1.68)	1.13 (0.94–1.35)	1.8%	1.92 (1.47–2.49)	1.54 (1.11–2.16)
Middle	40.4%	1.45 (1.36–1.54)	1.16 (1.08–1.24)	7.0%	2.26 (1.97–2.58)	1.37 (1.15–1.63)	3.3%	3.57 (2.79–4.58)	1.93 (1.42–2.63)
Richer	53.9%	2.08 (1.95–2.22)	1.38 (1.28–1.48)	13.7%	4.43 (3.9–5.03)	1.98 (1.67–2.35)	6.8%	7.26 (5.74–9.19)	2.87 (2.13–3.87)
Richest	80.2%	4.99 (4.64–5.36)	2.42 (2.20–2.66)	39.6%	13.98 (12.40–15.77)	3.74 (3.10–4.50)	22.3%	23.54 (18.80–29.47)	5.80 (4.24–7.93)
**Maternal education**									
No education	26.7%	1	1	3.1%	1	1.00	1.2%	1	1
Primary	45.9%	1.97 (1.87–2.07)	1.43 (1.1.35–1.51)	8.8%	2.73 (2.44–3.06)	1.48 (1.27–1.73)	3.6%	2.81 (2.27–3.48)	1.39 (1.05–1.83)
Secondary	72.3%	4.50 (4.73–5.28)	2.41 (2.24–2.64)	25.1%	9.11 (8.23–10.09)	2.24 (1.91–2.64)	12.8%	10.72 (8.85–12.99)	2.15 (1.64–2.80)
Higher	95.7%	33.14 (26.59–41.30)	9.68 (7.58–12.34)	65.9%	48.31 (42.34–55.12)	4.83 (3.84–6.06)	41.1%	47.99 (38.98–59.08)	3.49 (2.51–4.85)
**Paternal education**									
No education	31.5%	1	1	4.5%	1	1.00	1.8%	1	1
Primary	46.5%	1.57 (1.49–1.65)	1.09 (1.03–1.15)	9.2%	2.04 (1.84–2.26)	1.08 (0.94–1.24)	4.2%	2.41 (2.02–2.88)	1.19 (0.93–1.51)
Secondary	63.3%	2.75 (2.61–2.91)	1.33 (1.25–1.43)	20.2%	4.86 (4.42–5.33)	1.45 (1.27–2.67)	11.2%	6.36 (5.41–7.48)	1.69 (1.34–2.13)
Higher	83.7%	6.85 (6.25–7.52)	1.63 (1.45–1.84)	47.8%	16.89 (14.89–18.20)	2.30 (1.94–2.73)	30.2%	21.03 (17.85–24.78)	2.62 (2.01–3.43)
**Age group of the mother**									
10–19	50.2%	1	1	13.0%	1	1.00	6.0%	1	1
20–29	51.1%	1.13 (1.07–1.19)	1.25 (1.18–1.39)	14.8%	1.13 (1.03–1.23)	1.24 (1.09–1.40)	8.5%	1.44 (1.25–1.66)	1.66 (1.37–2.00)
30–39	43.7%	0.8 (0.75–0.86)	1.26 (1.34–1.68)	14.0%	1.00 (0.90–1.11)	2.09 (1.73–2.54)	9.1%	1.41 (1.21–1.66)	3.44 (2.60–4.56)
≥40	30.4%	0.44 (0.38–0.50)	0.92 (0.78–1.07)	6.2%	0.44 (0.34–0.58)	2.12 (1.46–3.08)	3.7%	0.56 (0.37–0.84)	3.99 (2.15–7.39)
**Birth order**									
1	63.7%	1	1	25.0%	1	1.00	15.3%	1	1
2	53.5%	0.96 (0.91–1.02)	0.95 (0.89–1.01)	17.0%	0.61 (0.56–0.65)	0.63 (0.57–0.70)	9.8%	0.58 (0.52–0.64)	0.57 (0.49–0.66)
3	46.3%	0.77 (0.72–0.82)	0.77 (0.72–0.83)	11.3%	0.37 (0.34–0.41)	0.47 (0.40–0.54)	5.6%	0.33 (0.29–0.38)	0.36 (0.29–0.46)
4	38.2%	0.60 (0.56–0.65)	0.63 (0.59–0.69)	6.4%	0.21 (0.19–0.24)	0.34 (0.28–0.42)	2.8%	0.18 (0.14–0.22)	0.25 (0.18–0.35)
5	33.6%	0.51 (0.47–0.56)	0.57 (0.51–0.63)	4.9%	0.14 (0.11–0.17)	0.26 (0.19–0.34)	1.3%	0.06 (0.04–0.09)	0.10 (0.06–0.16)
6 and above	26.5%	0.35 (0.33–0.38)	0.41 (0.37–0.46)	3.1%	0.08 (0.07–0.10)	0.20 (0.15–0.28)	1.6%	0.05 (0.03–0.07)	0.1 (0.05–0.20)
**Religion**									
Islam	48.0%	1	1	13.3%	1	1.00	7.9%	1	1
Hindu and others	55.0%	1.14 (1.06–1.22)	1.10 (1.01–1.19)	21.2%	1.70 (1.55–1.86)	1.73 (1.48–2.02)	10.6%	1.43 (1.24–1.64)	1.28 (1.02–1.61)
**Division**									
Barisal	50.8%	1	1	8.3%	1	1.00	4.4%	1	1
Chittagong	53.9%	1.13 (1.03–1.24)	1.09 (0.99–1.13)	9.9%	1.23 (1.04–1.44)	1.03 (0.83–1.27)	6.0%	1.39 (1.08–1.79)	1.15 (0.88–1.51)
Dhaka	54.6%	1.17 (1.07–1.27)	1.07 (0.8–1.24)	14.4%	1.87 (1.60–2.18)	1.44 (1.18–1.75)	9.3%	2.25 (1.77–2.86)	1.78 (1.36–2.33)
Khulna	58.3%	1.35 (1.22–1.5)	1.2 (1.07–1.35)	19.3%	2.65 (2.24–3.13)	2.23 (1.81–2.76)	10.6%	2.57 (1.98–3.34)	1.92 (1.46–2.52)
Rajshahi	53.5%	1.11 (1.02–1.22)	1.29 (1.17–1.44)	10.2%	1.27 (1.07–1.49)	1.53 (1.24–1.88)	5.3%	1.23 (0.95–1.59)	1.43 (1.08–1.89)
Sylhet	60.5%	1.48 (1.33–1.65)	1.45 (1.28–1.63)	11.5%	1.44 (1.20–1.73)	1.43 (1.15–1.79)	5.3%	1.22 (0.91–1.62)	1.31 (0.97–1.75)
Rangpur	62.9	2.64 (1.38–1.95)	0.996 (0.82–1.21)	19.2%	2.63 (2.07–3.35)	1.34 (0.98–1.82)	11.1%	2.73 (1.96–3.81)	1.52 (1.08–2.15)
**Area of residence**									
Rural	40.6%	1	1	8.2%	1	1.00	5.0%	1	1
Urban	72.0%	3.18 (2.99–3.37)	1.71 ((1.60–1.84)	31.0%	5.02 (4.70–5.35)	2.13 (1.88–2.42)	15.5%	3.69 (3.37–4.05)	1.52 (1.08–2.15)

**Fig 2:**
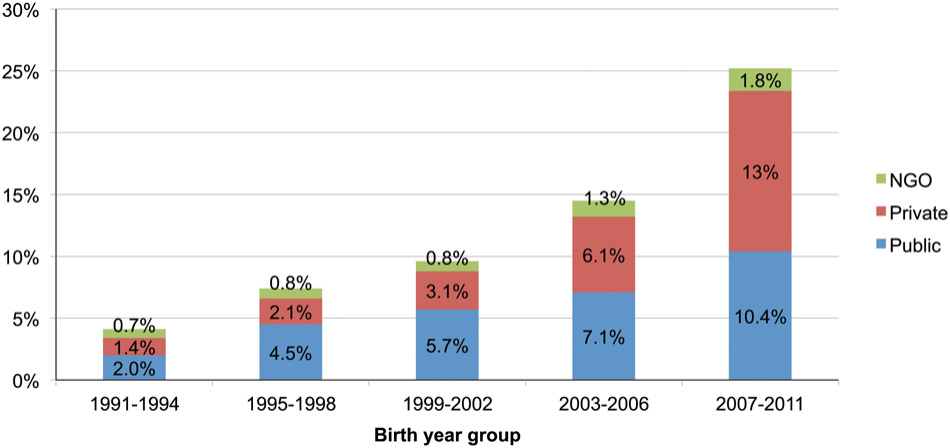
Distribution of place of delivery over time.

Fig. 2 shows that facility delivery rate increased both in public and private sector facilities. However, the rate of increase was higher for-profit private facilities than in public facilities. During 1991–1994, only 1.4% of deliveries were conducted in for-profit private sector health facilities which increased to 13.0% during 2007–2011; while the same increase was from 2.0% to 10.4% in public sector health facilities. Fewer deliveries took place in NGO facilities (Fig. 2) and about two third of deliveries still took place at home, mostly by untrained attendants.

### Trends in inequities in maternity care services

**Fig 3:**
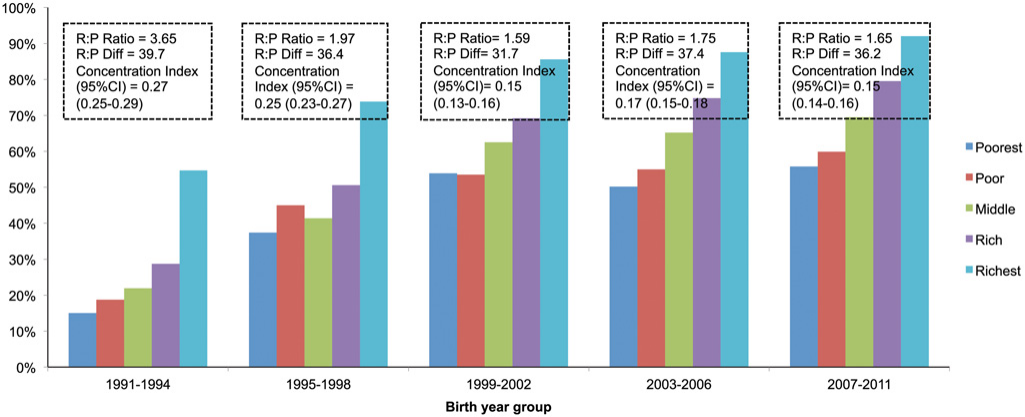
Inequity in use of antenatal care over time.

**Fig 4:**
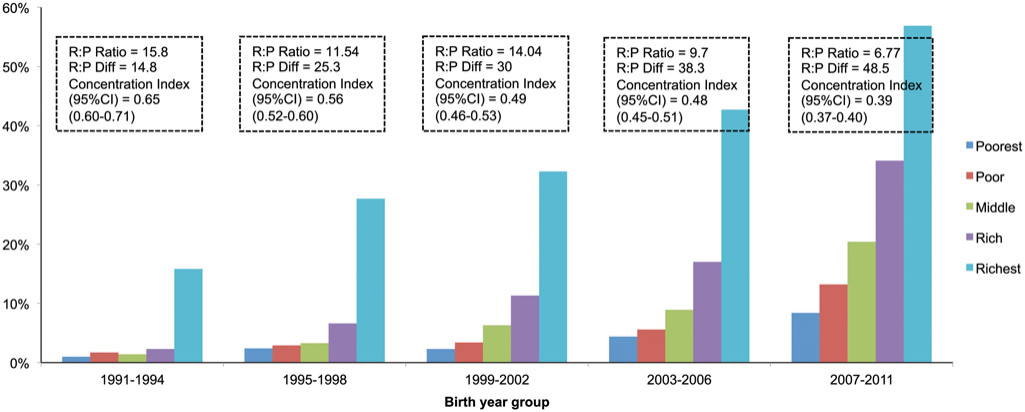
Inequity in use of facility delivery over time.

**Fig 5:**
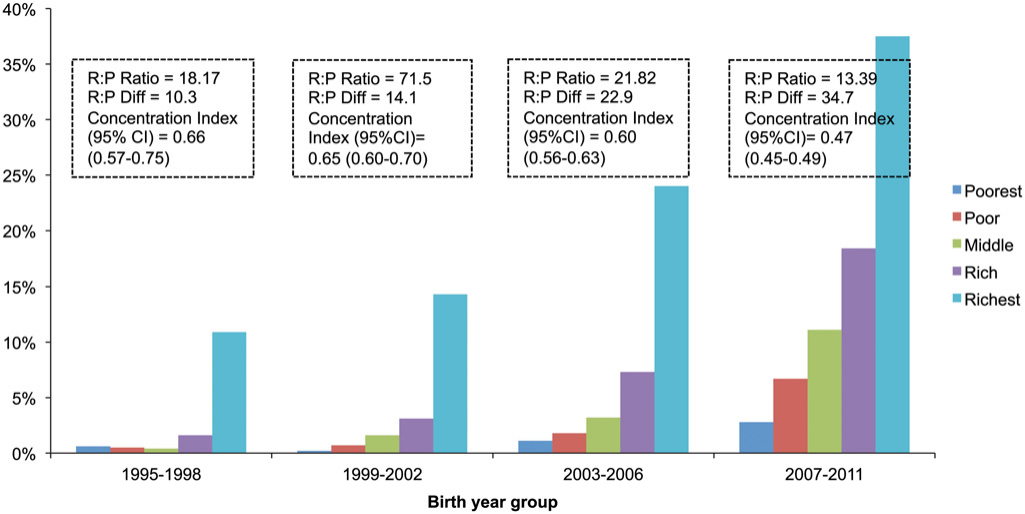
Inequity in use of C-Section over time.

There was use-inequity in all three maternal health process indicators but inequity was more important for C-section delivery than institutional delivery or ANC use (Figs. 3–5 and Table 3). Adjusted odds ratios (ORs) between the richest and the poorest quintile mothers for use of ANC, facility delivery and C-section delivery were 2.42 (95%CI: 2.20–2.66), 3.74 (95% CI: 3.10–4.50), and 5.80 (95% CI: 4.24–7.93) respectively (Table 3). However, our analysis suggests that inequity is reducing over time in terms of rich:poor ratio and concentration indices (Figs. 3–5). For the use of ANC services, the rich:poor ratio reduced from 3.65 in 1991–1994 to 1.65 in 2007–2011 and the concentration index from 0.27 (0.25–0.29) to 0.15 (0.14–0.16). For institutional delivery, the rich:poor ratio reduced from 15.80 to 6.77 and the concentration index from 0.65 (0.60–0.71) to 0.39 (0.37–0.41) between 1991–1994 and 2007–2011. For C-section deliveries, the rich:poor ratio reduced from 18.17 to 13.39 and the concentration index from 0.66 (0.57–0.75) to 0.47 (0.45–0.49) between periods 1995–1998 and 2007–2011 respectively. However, when measured in terms of rich:poor differences (in percent points), there was an equity-gain for the use of ANC services over time but not for facility delivery or C-section delivery (Figs. 4–5).

Table 2 shows that the yearly increase of facility delivery and C-section delivery rate was higher among the poorest mothers than their richest counterparts although the difference was not statistically significant. For C-section delivery, the yearly increase rate was significantly higher among middle class women than women from other economic groups (Table 2). For ANC services, the yearly increase of use was highest amongst higher socioeconomic group mothers.

Use-inequality was present by area of residence as well: women from urban areas were more likely to receive ANC (OR 1.71; 95% CI: 1.60–1.84), have institutional delivery (OR 2.13; 95% CI 1.88–2.42) and to deliver by C-section (OR 1.52; 95% CI: 1.08–2.15). It is important to note that the yearly trend for the use of ANC services was 9.4% (95% CI 8.9%-9.9%) in rural areas compared to 6.3% (95% CI 5.0%-7.6%) in urban areas. The yearly increase in facility delivery rate was 14.1% (95% CI 13.1%-15.1%) in rural areas compared to 8.3% (95% CI 7.0%-9.7%) in urban areas; and for C-section delivery, the adjusted yearly increase was 23.2% (95% CI 21.0%-25.5%) in rural areas and 13.9% (95% CI 11.7%-16.3%) in urban areas (Table 2). Data suggests urban-rural differentials in use of maternal health care services are reducing over time.

### Socio-demographic determinants of use of maternity care services

In unadjusted analysis, all socio-demographic variables were significant predictors of use of ANC, institutional delivery and C-section delivery (Table 3). Utilization varied significantly by asset quintile, parental education, and religion, area of residence, division, and age-group and parity of mothers. Consistent with the unadjusted analysis, in multivariable analysis, when the effects of covariates were controlled statistically using binary logistic regression model in SPSS 20, all socio-demographic factors remained significant predictors of use of ANC, institutional delivery and C-section delivery. Among socio-demographic variables, maternal education was the most powerful predictor for all three outcome (use) variables. Compared to mothers without any education, mothers with higher education were about ten times more likely (OR: 9.68; 95% CI: 7.58–12.34) to use antenatal care, five times more likely (OR: 4.83; 95% CI: 3.84–6.06) to deliver at the facility, and almost 3.5 times more likely (OR: 3.49; 95% CI: 2.51–4.85) to deliver their babies by C-section (Table 3). Asset quintile was the second most powerful predictor for institutional delivery and C-section delivery and third most powerful predictor for use of ANC services.

In bivariate analysis, the use of maternity care services was found to decrease with increasing age. While in multivariate analysis, the use of maternity care services was found to increase with increasing age. Birth order showed a consistent negative association with the use of maternity care services. The odds for use of all maternity care services decreased with increasing birth order (Table 3). Religion was the weakest (but significant) predictor for use of ANC services, institutional delivery and delivery by C-section. Mothers from religious minority groups were 10%, 70% and 28% more likely to use ANC services, facility delivery and c-section delivery respectively than their Muslim counterparts.

Data suggests significant regional variation in the use of maternity care services with poorer performance in Sylhet, Barisal and Chittagong divisions and better in Khulna, Dhaka and Rangpur divisions (Table 3).

## Discussion

The study aimed to explore the trends, inequities and other socio-demographic determinants of the use of maternity care services in Bangladesh between 1991 and 2011. Findings showed that substantial progress has been made, both in increased use and reduced use-inequity in maternal health over the last twenty years. However, gain in access to institutional delivery has been slow and attributed mainly to the for-profit private sector in health. The rising C-section rate is alarming, particularly among urban, wealthy and educated women. Regional variation in access to maternal health care services is evident. Maternal education is the most powerful predictor of use among all socio-demographic factors examined. The social determinants of health have also improved considerably over the last two decades in Bangladesh, particularly female education. Results indicated that educational gain was greater for women than their husbands. Demographic transition is also apparent in a substantial shift downwards in terms of parity distribution although the rate of teenage pregnancy remains high.

There has been a significant increase in the use of ANC services, institutional delivery and C-section delivery rates in Bangladesh during the last two decades. However, the rate of increase was higher for C-sections than for institutional deliveries or any ANC uptake. The institutional delivery rate increased from 4.3% during 1991–1994 to 33.9% in 2007–2011. With the current rate of increase, it will be difficult for Bangladesh to achieve its national target of 50% institutional delivery by 2015. The increase in institutional delivery is mainly attributed to the for-profit private sector facilities due to a complex of factors including demand, convenience and revenue. A similar situation exists in neighboring India where some states are expected to achieve high institutional delivery proportions with two third of all births occurring in the private sector by 2020 [21]. Over the past two decades, there has been a rapid increase in prominence of the private sector in health in Bangladesh [22], including maternal healthcare services [7]. The literature suggests that users consider private services to be of better quality than services from public facilities as they are more responsive, have shorter waiting times, and ensure greater confidentiality [23]. However, private sector providers is unregulated and there are concerns about the quality of care given and the associated costs involved. Users are poorly informed of their health care needs and unable to judge many aspects of quality and cost; thus many are vulnerable to poor quality services and over-charging. Given the importance and rapid expansion of the private sector, there is an urgent need to better understand their motivations and care practices, to engage them more productively in the health system and to ameliorate growing use-inequities for better health outcomes.

The rapid increase in population based C-section rate is obvious and is a growing concern. WHO/UNICEF recommend a population based C-section rate between 5% and 15% [24]. In 2011, the all cause C-section rate was 19.8% in Bangladesh, considerably higher than global recommendation. The rate of C-section is significantly higher among women with greater education, women from urban areas and from the richest quintile households. On the contrary, the rate is lower than the minimum recommended level of 5% among women from the poorest quintile households and among women with no education. This raises the question of whether these costly and life-saving surgeries are being conducted for women who really need them. Both increased demand and supply are likely responsible for this situation. Recent data from multiple settings suggest that rich and educated women may prefer caesarean section to vaginal delivery because they believe it to be safer [25, 26]. Supply side factors might have been playing role as quick C-sections interfere less with the workload or leisure time of the service providers. Excessive C-sections could also be due to perverse incentive mechanisms attached to the procedure. In addition, global data suggests that increased availability of resources from innovative health financing strategies [27] have contributed to increasing C-section rates in different settings [28, 29]. The ongoing demand side financing (DSF) maternal health voucher scheme in Bangladesh might have some role in the increasing population based C-section rates [11]. However, DSF is only available in 53 rural sub-districts out of the total 490 sub-districts. Thus, their contribution in increasing C-section rates, if any, should be minimal. Experts have cautioned that “C-sections, unless strictly indicated, may be harmful to the health of mothers and their newborn babies” [30]. In this regard, there is a need to monitor the indication of C-sections in both public and private facilities as suggested by Stanton et.al [26]. Advocacy to key medical and public health professionals is also required.

Exploring use-inequity was a key objective of the study. Our analysis indicates significant use-inequity for all three maternal health process indicators by asset quintile, parental education and area of residence. However, inequity was higher for higher level emergency obstetric care services (C-section) than lower-level preventive services (such as use of ANC and institutional delivery). Bangladesh data suggests improvement in access-equity for ANC services, institutional delivery and C-section deliveries over time in terms of rich: poor ratio and concentration indices. However, when using measures of absolute difference between richest and poorest, there was an equity gain for ANC services over time but not for institutional delivery or population based caesarean section rates. This suggests that it is important to decide which measures health systems managers should apply on a day-to-day basis to monitor access inequity. The literature suggests that concentration indices and curves are better measures as they take into account all socio-economic groups in the calculation. Other measures such as rich:poor ratio or rich:poor difference, take into account only the richest and poorest quintile mothers in the calculations. Global data suggests that service coverage and use-inequity are interrelated. In settings where service coverage is high, the use-inequity in general remains low, as services are accessible by the majority of the population, regardless of socio-economic group. Similarly, generalized low coverage may also result in low inequity as the majority of the population groups have the same low level of use [31]. Therefore, when interpreting equity analysis findings, it is important to take into account the overall coverage.

In Bangladesh, a number of pro-poor health and non-health interventions are being run by the government, NGOs and development partners to improve social determinants of health. The important ones are the DSF maternal health voucher program in the health sector [11] as has been mentioned earlier and the food for education program for poor female students in the education sector. Microcredit programs have contributed to relatively better access gain to essential health care services by the poor. Our findings support the view expressed in the Lancet Bangladesh series that socio structural factors have contributed in improvements in health indicator status and equity [32, 33]. However, recent literature suggests that there are certain potential challenges in the health sector in Bangladesh, such as poor and ineffective governance, weak regulatory framework and lack of trained human resources particularly in remote, rural and hard to reach areas [34].

Despite these challenges, the country has reduced its MMR over the last two decades and this reduction took place with little improvement in facility delivery or skilled attendance rates. During 2013, WHO reported an MMR of 170 deaths per 100,000 live-births in Bangladesh and an MMR of 190 deaths per 100,000 live-births in Indonesia [35] despite much higher rates of institutional delivery in Indonesia than Bangladesh. This raises question about the validity of facility delivery rate (or skilled attendance rate) as a proxy for MMR. Anecdotal data suggests that although Bangladeshi women prefer to deliver at home, they have a contingency plan to visit emergency obstetric care facilities if complications arise and can overcome access-barriers through social networks [36]. Our data is in accordance with this view. Recent improvements in social determinants of health, mobile and road transport communication, along with the establishment of many private sector hospitals throughout the country, have contributed to improved access to comprehensive emergency obstetric care (CEmOC) services. Bangladesh data supports the view expressed by others that those who conduct deliveries is important, but more important is whether women can access emergency live-saving obstetric care services when and if complication arises [37].

Our analysis indicates that all socio-demographic factors examined are significant predictors for the use of maternity care services. However, their strength of association with outcome variables varied. Among all socio-demographic factors, maternal education emerged as the number one predictor of use of maternity care services. Studies in other settings have also explored the influence of maternal education on the use of maternity care services [30]. Experts have opined that in low-income settings more emphasis should be given to female education than other social determinants of health [33]. The effect of demographic variables on the use of services is also significant. In bivariate analysis, it appears that the utilization rate decreases with increasing age. This is an artifact due to confounding effect of parity; when parity remains constant, the probability of use of maternity care services increases with increasing age.

The study has certain limitations. First, recall period varied in the six BDHS surveys; during the 1993–1994 survey, the recall period was 3 years while in other surveys it was 5 years. We argue that the disadvantage of different recall periods would be offset by the gain in power obtained by aggregating all available data. Second, with rapid urbanization that is occurring in Bangladesh, there is a change in the definition of urban and rural areas over time. As a consequence, some areas which were classified as rural in the earlier BDHSs were considered urban in the more recent BDHSs, and this may introduce some error in urban-rural calculations. Rangpur is a new division created from the Rajshahi division in 2010 and this information is available only in the 2011 BDHS. In earlier surveys, mothers from the Rangpur area were shown as mothers from the Rajshahi division.

## Conclusion

In order to end preventable maternal mortality, it is essential to strengthen universal health coverage and access-equity in maternal health. Ongoing pro-poor health and non-health interventions should be strengthened, and more innovative health financing strategies should be tested under the newly formulated ‘Expanding Social Protection for Health Towards Universal Coverage: Health Care Financing Strategy 2012–2032’ [38]. Routine monitoring of use-inequity in maternal health using multiple indicators deserves special attention. There is also a need to develop a routine system to monitor indication of C-sections in both public and private sector health facilities to rationalize its use in improving maternal health outcomes and in reducing health systems’ costs.
